# Automatic post-stroke lesion segmentation on MR images using 3D residual convolutional neural network

**DOI:** 10.1016/j.nicl.2020.102276

**Published:** 2020-05-26

**Authors:** Naofumi Tomita, Steven Jiang, Matthew E. Maeder, Saeed Hassanpour

**Affiliations:** a Department of Biomedical Data Science, Geisel School of Medicine at Dartmouth, Hanover, NH 03755, USA; b Department of Computer Science, Dartmouth College, Hanover, NH 03755, USA; c Department of Radiology, Dartmouth-Hitchcock Medical Center, Lebanon, NH 03756, USA; d Department of Epidemiology, Geisel School of Medicine at Dartmouth, Hanover, NH 03755, USA

**Keywords:** CNN, convolutional neural network, 3D, three dimensional, T1W, T1-weighted, DSC, Dice similarity coefficient, ASSD, average surface symmetric distance, HD, Hausdorff distance, Ischemic stroke, Segmentation, Deep learning, MRI

## Abstract

In this paper, we demonstrate the feasibility and performance of deep residual neural networks for volumetric segmentation of irreversibly damaged brain tissue lesions on T1-weighted MRI scans for chronic stroke patients. A total of 239 T1-weighted MRI scans of chronic ischemic stroke patients from a public dataset were retrospectively analyzed by 3D deep convolutional segmentation models with residual learning, using a novel zoom-in&out strategy. Dice similarity coefficient (DSC), average symmetric surface distance (ASSD), and Hausdorff distance (HD) of the identified lesions were measured by using manual tracing of lesions as the reference standard. Bootstrapping was employed for all metrics to estimate 95% confidence intervals. The models were assessed on a test set of 31 scans. The average DSC was 0.64 (0.51–0.76) with a median of 0.78. ASSD and HD were 3.6 mm (1.7–6.2 mm) and 20.4 mm (10.0–33.3 mm), respectively. The latest deep learning architecture and techniques were applied with 3D segmentation on MRI scans and demonstrated effectiveness for volumetric segmentation of chronic ischemic stroke lesions.

## Highlights

• We developed a deep learning model for MRI 3D lesion segmentation of chronic stroke.
• Our novel zoom-in&out strategy increases performance and accelerates optimization.
• High performance was achieved in both volumetric and surface-based metrics.

## Graphical abstract

**Figure:**
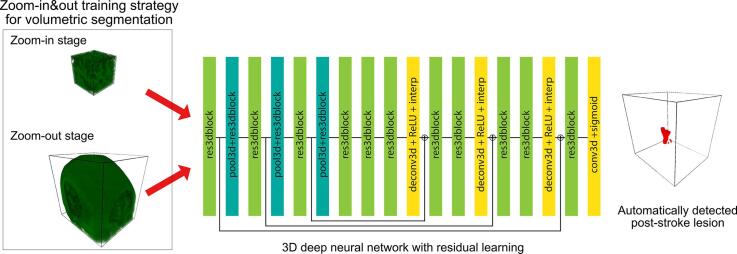

## 1 Introduction

Stroke is one of the leading causes of long-term adult disability worldwide (Mozaffarian et al., 2015). Recent studies show that 36% to 71% of post-stroke survivors had a disability after at least five years (Hankey et al., 2002, Hardie et al., 2004, Jönsson et al., 2014, Yang et al., 2016) . Rehabilitation is crucial for long-term functional recovery. The effectiveness of rehabilitation varies, however, because functional and structural changes in the brain differ among patients. Identifying the damaged brain network in patients would help clinicians to predict functional outcomes in response to targeted rehabilitation, which benefits patients by optimizing treatment resources and providing personal and efficient care (Burke Quinlan et al., 2015, Marie-Héléne and Cramer, 2008, Riley et al., 2011) . T1-weighted (T1W) magnetic resonance imaging (MRI) is the most common resource used in research for chronic stroke lesions because lesions are visible on T1W images after a month and the produced images have high resolution. Tracing these lesions manually, however, is time intensive and prone to errors (Fiez et al., 2000).

Many approaches have been proposed for automatic segmentation of chronic lesions on T1W MRIs after a stroke (Seghier et al., 2008, Wilke et al., 2011, Mitra et al., 2014, Pustina et al., 2016, Detante and Dojat, 2017, Yang et al., 2019, Qi et al., 2019, Zhou et al., 2019, Fadi et al., 2020). Compared to research on automatic segmentation of acute stroke lesions, however, methods for chronic lesion segmentation are underdeveloped. One major difference between acute and chronic lesions in terms of image segmentation is that the former utilize diffusion—and/or perfusion—weighted imaging, while the latter typically use high-resolution T1W imaging. Methods developed for acute stroke lesion segmentation are not readily applicable to chronic stroke analysis due to the different characteristics of these MRI pulse sequences and the high-resolution data of T1W MRIs.

Recently, convolutional neural networks (CNNs) have achieved expert-level performance in various radiology image analysis tasks (Larson et al., 2017, Tomita and Cheung, 2018, Becker et al., 2018, Liu et al., 2019) . Three-dimensional (3D) CNNs are deep learning architectures that can extract 3D spatial features. Since diagnosing stroke lesions by neuroradiologists requires analysis of a lesion and its surrounding area (Crinion et al., 2013), 3D CNNs are suitable for this task. This is because 3D CNNs incorporate the contextual information of voxels (i.e., volumetric pixels) into analysis by capturing both low-level local features (i.e., edges and corners) and high-level global features (i.e., the anatomy of brains).

In this study, we developed an effective deep learning model for 3D segmentation to identify areas of infarcted brain tissue on MRI images. To develop our method, we utilized a public dataset of T1W MRI scans from patients with chronic stroke lesions.

## 2 Materials and methods

### 2.1 Data source

To develop and evaluate our algorithm in this study, we used a publicly available dataset of volumetric MRI scans of patient brains with anatomical tracings of lesions after stroke (ATLAS) (Liew et al., 2017). In the ATLAS dataset, a total of 304 MRI scans were collected. Stroke lesions on T1-weighted MRI images were manually traced and established by trained students and research fellows under the supervision of an expert tracer and a neuroradiologist. The collection of the ATLAS dataset and the subsequent sharing of the data were approved by the study’s Institutional Review Board (IRB). Informed consent was obtained from all subjects before data collection. We used a subset of the ATLAS dataset, which consists of 239 scans normalized to MNI-152 space (Liew et al., 2017). The size of scans is 197 × 233 × 189 mm^3^ and the canonical size of a voxel is 1 mm^3^. Lesion size in the dataset ranges from 10 mm^3^ to 2.8 × 10^5^ mm^3^. Demographic data of the dataset is not available. The statistics of the dataset are summarized in Tables E1, E2, E3, and E4 in the Supplementary Material.

### 2.2 3D segmentation using a deep convolutional neural network

**Fig. 1:**
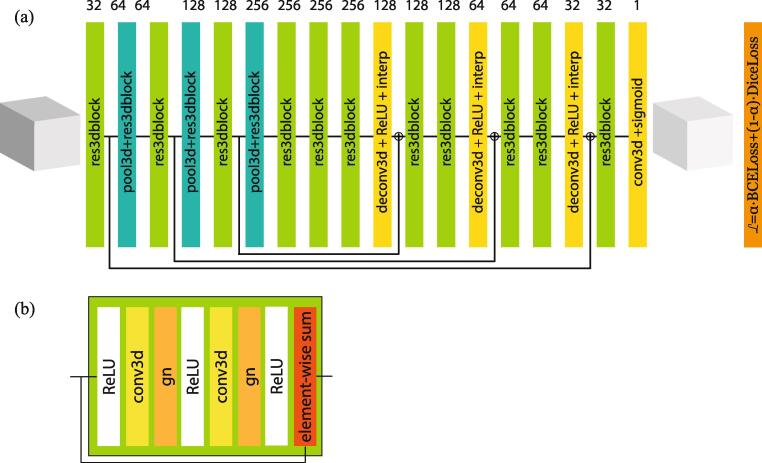
Overview of our 52-layer segmentation model. (a) The network consists of residual blocks (in green), down-sampling blocks (in blue), and up-sampling blocks (in yellow). (2) Our 3D residual block uses a group-normalization (gn) layer to stabilize optimization for a small mini-batch. (For interpretation of the references to color in this figure legend, the reader is referred to the web version of this article.)

For the 3D brain lesion segmentation task, we use a 3D U-Net (Çiçek et al., 2016), which is the state-of-the-art deep learning architecture for volumetric segmentation tasks. U-Net architecture has characteristic internal skipping connections between layers to propagate information from earlier layers (encoder) to later layers (decoder). Fig. 1 shows the overview of our 3D U-Net model in this study. We extended the 3D U-Net architecture to accommodate our task and we detailed the modification in the Supplementary Material, Appendix E1. Our objective function *L* is an affine combination of the binary cross entropy (BCE) loss function and the Dice loss function (Milletari et al., 2016), which we describe in detail in the Supplementary Material, Appendix E2.

### 2.3 Zoom-in&out training strategy

To efficiently train our models^1^, we used a two-stage zoom-in&out strategy to first train them on small volumes, and then we finetuned the models on larger volumes. This two-stage training has multiple advantages. First, training models with smaller volumes can have a regularizing effect due to performing data augmentation by randomly extracting diverse sub-volumes from original volumes. Second, a “zoom-in” step is a computationally inexpensive option and can utilize sub-optimal graphic processing units (GPUs) for the task. By feeding smaller volumes to older but more accessible GPUs, models can be trained in parallel, and, as a result, are faster. Finally, the “zoom-out” stage involves showing models larger volumes to learn from the broader context of input images and improves the robustness of the model.

### 2.4 Experimental settings

The dataset was split into training, development, and test sets, containing 182; 26; and 31 MRI exams, respectively. In Table 1, we summarized the details and selected hyperparameters for the zoom-in&out stages of the optimization. During evaluation, we cropped out a center volume (144 × 172 × 168 mm^3^) from the whole MRI scans and fed it to the network to make predictions. All the voxels outside of the cropping window were automatically classified as negative. In addition to our final model at the 150th epoch of finetuning, we built a snapshot ensemble of models at the 50th, 100th, and 150th epoch of the zoom-out stage (Huang et al., 2017). 3D-ResU-Net and 3D-ResU-Net-E denote, respectively, the final model and the snapshot ensemble model. For reproducibility, the complete list of subject IDs in each split is shown in Table E5 in the Supplementary Material.

**Table 1:** The details and hyperparameters for the model optimization in our experiments. Additional details about the input volume selection are available in Appendix E3.

Optimization Stage	Zoom-In Stage	Zoom-Out Stage
Input volume size (mm^3^)	128 × 128 × 128 (24% sub-volume)	144 × 172 × 168 (48% sub-volume)
Training Length	1200 epochs	150 epochs
Initial learning rate	1.00E−03	1.00E−04
Optimizer	Adam optimizer and cosine annealing with warm restart scheduler (Pustina et al., 2016, Qi et al., 2019)	
GPU	Nvidia Titan Xp with 12 GB memory	Nvidia Titan RTX with 24 GB memory
Deep learning framework	PyTorch (Paszke et al., 2017)	

### 2.5 Evaluation metrics

We evaluated the performance of our segmentation methods on the test set by computing the Dice similarity coefficient (DSC), maximal DSC (mDSC), Hausdorff distance (HD), average symmetric surface distance (ASSD), true positive rate (TPR), and precision for each MRI scan. DSC, HD, and ASSD were computed using a surface distance computation library (DeepMind, 2018). TPR and precision were computed by using the scikit-learn package version 0.21.1 (Pedregosa et al., 2011), and mDC was implemented according to the algorithm (Chen et al., 2018). To estimate 95% confidence intervals, we employed bootstrapping with 1000 iterations for all metrics.

## 3 Results

### 3.1 Prediction performance

Evaluation metrics of our model are summarized in Table 2. 3D-ResU-Net yields an average DSC of 0.64 (0.51–0.74), maximal DSC of 0.66 (0.54–0.76), HD of 20.4 mm (10.0–33.3 mm), ASSD of 3.6 mm (1.7–6.2 mm), TPR of 0.81 (0.68–0.90), and precision of 0.62 (0.48–0.74). For 3D-ResU-Net-E, the performance is an average DSC of 0.64 (0.51–0.76), maximal DSC of 0.65 (0.53–0.77), HD of 21.5 mm (10.0–33.9 mm), ASSD of 3.7 mm (1.6–6.3 mm), TPR of 0.79 (0.67–0.89), and precision of 0.63 (0.48–0.75). Human inter-rater scores are also presented in the last row in Table 2 as a reference. Unlike previous work (Huang et al., 2017), the ensemble of our snapshots did not improve the model’s performance. Following these results, we used 3D-ResU-Net for further experiments.

**Table 2:** Summary of evaluation metrics. A higher rate is better for DSC, mDSC, TPR, and Precision. For distance metrics (HD and ASSD), a smaller number is better. Best scores are marked in bold. The inter-rater scores are calculated based on tracing of five brain MRIs by 11 non-expert individuals trained by an expert neuroradiologist (Liew et al., 2018). The model’s performance, based on the primary stroke locations and the vascular territories, is available in the Supplementary Material, Tables E6 and E7.

Methods	DSC	mDSC	HD (mm)	ASSD (mm)	TPR	Precision
3D-ResU-Net	0.64 (0.51–0.76)	**0.66** **(0.54**–**0.76)**	**20.4** **(10.0**–**33.3)**	**3.6** **(1.7**–**6.2)**	**0.81** **(0.68**–**0.90)**	0.62 (0.48–0.74)
3D-ResU-Net-E	0.64 (0.51–0.76)	0.65 (0.53–0.77)	21.5 (10.0–33.9)	3.7 (1.6–6.3)	0.79 (0.67–0.89)	**0.63** **(0.48**–**0.75)**
Trained human tracer	**0.73** **(0.53**–**0.93)**	–	22.6 (1.2–43.9)	–	–	**–**

### 3.2 Comparison with existing methods

We identified recent studies of automatic segmentation that were conducted on the ATLAS dataset and summarized them in Table 3. X-Net (Qi et al., 2019), D-UNet (Zhou et al., 2019), CLCI-Net (Yang et al., 2019), and our 3D-ResU-Net use specific subsets of the ATLAS data to train and test their models, while Multi-path 2.5D-CNN (Fadi et al., 2020) was trained with two other datasets and tested on the ATLAS dataset. All the models are based on either 2D or 3D U-Net architecture. Among the 3D U-Net based models, our 3D-ResU-Net model uses significantly larger input volume than that of D-UNet. Although Multi-path 2.5D-CNN takes much larger volume as input than 3D-ResU-Net does, the model does not fully utilize context information in 3D space since the model analyzes input data in 2D and aggregates the results from each slice in the axial plane through post-processing. Besides zoom-in&out, our model leverages recent technical advancement in both training strategy (i.e., a combination of loss functions and cosine learning rate annealing) and network architecture (i.e., 3D fully convolutional neural networks and group normalization).

**Table 3:** Summary of different approaches on the ATLAS dataset. LR: learning rate; H: height; W: width; D: depth; SGD: stochastic gradient descent. “–” denotes that the corresponding information is not available.

Methods	X-Net (Qi et al., 2019)	Multi-path 2.5D-CNN (Fadi et al., 2020)	D-UNet (Zhou et al., 2019)	CLCI-Net (Yang et al., 2019)	3D-ResU-Net (ours)
Training data source	ATLAS	KF & MCW	ATLAS	ATLAS	ATLAS
ATLAS split ratio (train, validation, test) (%)	5-fold cross-validation	(0, 0, 100)	(80, 20, 0)	(55, 18, 27)	(76, 11, 13)
Base architecture	2D U-Net	2D U-Net with 3D post-processing	3D U-Net	2D U-Net	3D U-Net
Regularization layers	Batch normalization	Batch normalization	Batch normalization	Batch normalization	Group normalization
Training strategy	Adam optimizer, reduce LR on plateau	SGD optimizer, exponential LR decay	SGD optimizer, constant LR	Adam optimizer, constant LR	Adam optimizer, cosine annealing
Loss function	Dice loss & Cross Entropy	Dice loss	Dice loss & Focal loss	Dice loss	Dice loss & Cross Entropy
Input size (W × H × D)	192 × 224 × 1	192 × 224 × 192	192 × 4 × 192	176 × 233 × 1	144 × 172 × 168
Reported DSC	0.49 (–)	0.54 (–)	0.54 (0.26–0.81)	0.58 (–)	0.64 (0.51–0.76)

### 3.3 Qualitative analysis

**Fig. 2:**
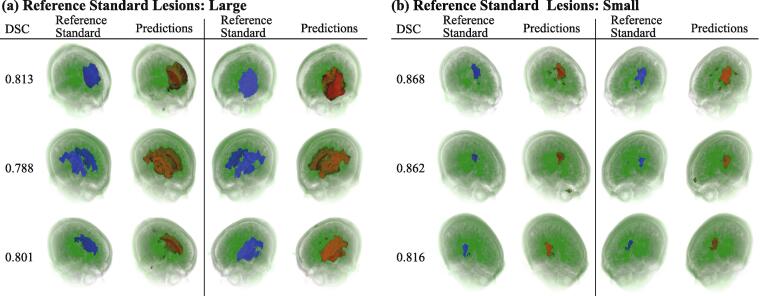
Visualization of reference standard labels (in blue) and lesion predictions by our model (in red). The higher the predicted value is at a voxel, the brighter in red the voxel is. Two groups of samples are shown: large reference labels in (a) and small labels in (b). For each group, the first column is a computed DSC value and the rest are visualized reference standards and predictions, from left-front and right-front views. Three typical samples are shown in a row in each group (best viewed in color). The visualization of segmentation results in axial slices is also available as videos in the Supplementary Material, Video E1. (For interpretation of the references to color in this figure legend, the reader is referred to the web version of this article.)

We visualized our automatic segmentation results on the test set by projecting voxel-wise predicted scores onto the original MRI volumes. Fig. 2 shows the visualization of reference standard labels and model output viewed from the front-left and front-right side of faces. Visualizations from other samples are also available in Fig. E1 in the Supplementary Material. The trained model accurately locates the chronic stroke lesions. Notably, while most of reference standard labels have an uneven structure on the surface, possibly due to the variability of manual human annotations, the predicted lesions tend to have smooth surfaces, which is a realistic assumption for such lesions. We hypothesize that the model has learned this continuous surface from data by internally averaging out marginal voxels of all the training cases and successfully removing variability in human annotation.

### 3.4 Lesion size and model performance

**Fig. 3:**
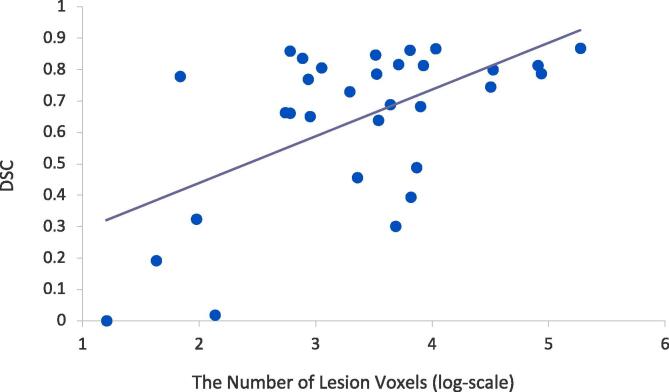
DSC scores and the total number of positive lesion voxels (in base-10 log scale) are computed and plotted for each sample in the test set. The R^2^ value of this distribution is 0.34.

We further analyzed the performance of our model in relation to the size of target lesions. Fig. 3 plots the number of positive voxels in the reference standard and a computed DSC of prediction for each sample in the test set. We observe a trend (R^2^ = 0.34; p-value <0.05) in which a sample with a large size of tracing has been predicted with a high DSC score. The median DSC is 0.75, which is 0.11 higher than the average. Table 4 shows a performance summary of the model given a subset of test samples where each subset is composed of a quarter of percentiles when samples are rank-ordered by the number of positive lesion voxels. For example, the first group includes test samples with the size of lesion voxels being smaller than the 25th percentile of the whole test set. The model achieves the highest DSC (0.74–0.84) and TPR (0.79–0.95) on samples with larger positive lesions (75%–100%). In the distance metrics, the model also achieves the lowest mean HD of 13.6 mm (2.8–35.2 mm) and mean ASSD of 1.8 mm (0.5–2.8 mm) for this group. We confirmed that segmentation performance in both voxel-based and surface-based metrics improves as the size of the lesion to be classified gets larger. The same trend is reported in Vorontsov et al., 2019, Ito et al., 2018. Of note, in our dataset, the median size of primary lesions is 3947 mm^3^ (interquartile range - IQR: [767 mm^3^, 21,639 mm^3^]). The median size of lesions, including secondary lesions, is 801 mm^3^ (IQR: [123 mm^3^; 6,049 mm^3^]). The median size of lesions that are aggregated per patient is 4,170 mm^3^ (IQR: [886 mm^3^; 21,639 mm^3^]).

**Table 4:** Comparison of evaluation metrics with respect to the subsets of test set samples. Test samples are sorted by the number of positive lesion voxels in increasing order and grouped in four ranges, shown in the first column. DSC, HD, ASSD, TPR, and precision are computed for each group. Best scores are marked in bold.

Percentile in per-sample lesion size distribution	DSC	HD (mm)	ASSD (mm)	TPR	Precision
0–25%	0.41 (0.07–0.78)	23.4 (3.7–51.1)	4.9 (0.7–13.2)	0.74 (0.30–0.99)	0.39 (0.01–0.81)
25–50%	0.72 (0.59–0.82)	18.0 (1.8–35.6)	3.8 (0.5–7.2)	0.78 (0.64–0.91)	0.71 (0.53–0.83)
50–75%	0.62 (0.42–0.80)	26.1 (2.3–58.0)	4.1 (0.6–8.8)	0.82 (0.53–0.96)	0.60 (0.39–0.79)
**75**–**100%**	**0.80** **(0.74**–**0.84)**	**13.6** **(2.8**–**35.2)**	**1.8** **(0.8**–**2.6)**	**0.87** **(0.79**–**0.95)**	**0.75** **(0.64**–**0.84)**

### 3.5 Effectiveness of zoom-in&out strategy

To further validate our methodology, we investigated the impact of our zoom-in&out training strategy on the performance of our model by evaluating models with and without finetuning on large volumes. In addition to the metrics we used for our main experiment, we computed the micro-average of DSC (microDSC), which is a global statistic used to evaluate the per-voxel performance of our model, and is less susceptible to the size of lesions. Here, 3D-ResU-Net-F denotes the model without the finetuning step, distinguished from the 3D-ResU-Net model. Table 5 summarizes this ablation study. Through finetuning, the per-voxel and per-sample segmentation performances are improved by 6% and 4%, respectively. The surface distances between the manual tracing and automatic segmentation measured by HD and ASSD are closer by 14.7 mm and 4.0 mm, respectively. Except for TPR, the model with larger volumes after finetuning shows higher performance across all metrics. Of note, training 3D-ResU-Net-F was converged after 1200 epochs with an annealed learning rate, thus we are confident that an additional 150 epochs of training does not improve the performance of the model without increasing the size of input volumes. Also, training models for 1200 epochs with the zoom-in&out method takes about 5 days, while training entirely with large volumes takes more than 3 weeks. Thus, the zoom-in&out strategy is an effective and viable option for training 3D segmentation models of large 3D T1W MRI images.

**Table 5:** Results of our ablation study examining the effect of our zoom-in&out training strategy. Finetuning with larger extracted volumes is applied on a 3D-ResU-Net-F model to obtain a 3D-ResU-Net model. The last row is the difference in performance between the 3D-ResU-Net and 3D-ResU-Net-F for each metric. Best scores are marked in bold.

Methods	microDSC	DSC	HD (mm)	ASSD (mm)	TPR	Precision
3D-ResU-Net-F	0.73	0.60 (0.47–0.73)	35.1 (20.4–51.3)	7.6 (3.7–12.3)	**0.83** **(0.71**–**0.91)**	0.54 (0.39–0.67)
3D-ResU-Net	**0.79**	**0.64** **(0.51**–**0.76)**	**20.4** **(10.0**–**33.3)**	**3.6** **(1.7**–**6.2)**	0.81 (0.68–0.89)	**0.62** **(0.48**–**0.74)**
Δ	+0.06	+0.04	−14.7	−4.0	−0.02	+0.08

## 4 Discussion

Identifying lesions and irreversible brain tissue damage on patient MRI scans after a stroke is challenging, especially when the amount of time and resources are limited. In this study, we developed a deep learning model for 3D segmentation of chronic stroke lesions to assist neuroradiologists in this task and further provide personalized rehabilitation for patients to achieve effective recovery. On the test set, the average symmetric surface distance of lesions identified by our segmentation model was 3.6 mm. The average Dice similarity coefficient score of our model was 0.64, with a median of 0.78. The overall performance of our model indicates that a 3D deep neural network is a promising method for volumetric segmentation of chronic stroke lesions on T1W MRI scans.

Our technical contribution in this study is twofold. First, we have established another baseline on the ATLAS dataset using the latest deep learning architecture and techniques to further encourage research in MRI analysis of chronic stroke patients. Second, we have presented a novel zoom-in&out strategy for effectively training 3D segmentation models on high-resolution volumes.

Recently, a patch-to-image training framework was proposed for segmentation of 2D fundus photographs. In that approach, a model is trained on extracted small patches from images in a dataset, and then it is finetuned on full-size images (Sekou et al., 2019). This procedure can be considered as a special case of our zoom-in&out strategy, where the previous work chose to feed the full-size input image at the zoom-out stage, while our method does not require the full-size input at the zoom-out stage, providing more flexibility in comparison to feeding the full images. Particularly, our approach is more suitable and essential for 3D image analysis that has an enormous GPU memory demand for training deep learning models. Using full-size input in the zoom-out stage would lose spatial variations in input samples and thus could lead to overfitting in model training, especially for 3D segmentation models with a large number of parameters. Conversely, randomly-extracted cropped images in our approach maintain the spatial variation of input and stabilize the training as they contribute as a regularizer in training 3D segmentation models. Of note, volumes in the zoom-out stage only account for 48% of the original scan size. MRI scans in our dataset are well-positioned and stretched in the normalized space; thus, our random crops still maintain spatial variation in foreground objects.

Of note, in our preliminary study, we explored the effect of the zoom-in stage’s input size on the model’s performance by considering 16 × 16 × 16 mm^3^ and 32 × 32 × 32 mm^3^ input volumes. The validation performance of the models trained with these smaller input volumes was not encouraging. Therefore, we considered larger input regions in training our model for post-stroke lesion segmentation. While we did not apply any random rotation as data augmentation in our study, training with small rotation may further benefit the model’s performance. In addition, we ran our model on test set samples stratified by scanners to investigate the effect of scanner variations on the model’s performance, and we could not see a statistical difference in the performance of our model based on scanner type.

This study has some limitations. Since the dataset is relatively small, further validations on external datasets of chronic stroke MRI scans are required to verify the generalizability of the model’s segmentation performance. We plan to investigate the generalizability of our model and its inter-scanner variance using larger multi-institutional datasets and scanner agnostic configurations in future work. The dataset used in this study contains only scans of embolic stroke, which accounts for the majority of strokes, however, further validation with other types of stroke is worthwhile. In addition, our method experienced the same problem as the previous work, in which segmentation performance degraded on volumes with small stroke lesions (Vorontsov et al., 2019, Ito et al., 2018). Small lesions are reasonably challenging to locate because the features of such lesion are subtle and hard to characterize. Notably, missing small lesions of primary stroke would result in a near-zero DSC score because the contribution of each positive voxel is much higher than that of cases with large lesions, and thus leads to having a much lower average DSC than median DSC score. We further measured per-patient lesion size characteristics for both the test and training sets. Interestingly, we found out the median lesion size of the test set (3328 mm^3^ [IQR: 730 mm^3^, 7888 mm^3^]) is smaller than that of the training set (4343 mm^3^ [995.5 mm^3^; 24,680.5 mm^3^]). Therefore, the test set with smaller lesions is a more challenging dataset to use for the evaluation of our model in comparison to cross-validation, in which the distributions of lesion size in the training and test sets are identical, validating the high performance of our model. Lastly, our error analysis shown in Fig. E2 (see Supplementary Material) demonstrates that a few MRI scans in the dataset have visual inconsistencies, possibly introduced at the time of original scanning or during image-processing steps in data curation. Our method does not require input scans to be in MNI-152 standard space and is applicable to datasets in native T1 space. However, we expect larger training sets are required for datasets without normalization to maintain the robustness of the trained models.

Currently, we are considering several avenues for extending our work. From a clinical perspective, lesion segmentation is a part of the clinical pipeline for providing rehabilitation service for stroke survivors. To fully extend the potential of current research for actionable clinical practice, we plan on building an application that 1) performs segmentation of chronic stroke lesions, 2) identifies disabled functionalities, and 3) predicts the effectiveness of rehabilitation for each neurological deficit, simultaneously. The first task provides evidence for the second task, and the second task forms a basis for the third task. This pipeline can provide a practical tool that aids clinical decision making. We expect 3D convolutional segmentation architectures are extendable to perform all three tasks. There is evidence that a multi-task model would learn robust features and achieve better performance than models that are trained for a single task (Fang et al., 2019, Samala et al., 2017) . To this end, another dataset that records the current status of disabilities, rehabilitation, and recovery of patients would be necessary, in addition to the MRI scans and segmentation masks, for our future work. We expect that our study will establish a standard in this domain and will promote further research to advance current state-of-the-art methodologies for volumetric segmentation of chronic ischemic stroke lesions on T1W MRI scans.

## Authorship contribution statement

**Naofumi Tomita:** Conceptualization, Methodology, Software, Validation, Formal analysis, Visualization, Investigation, Writing - original draft, Writing - review & editing. **Steven Jiang:** Conceptualization, Software, Writing - original draft. **Matthew E. Maeder:** Validation. **Saeed Hassanpour:** Conceptualization, Validation, Supervision, Resources.

## Declaration of Competing Interest

The authors declare that they have no known competing financial interests or personal relationships that could have appeared to influence the work reported in this paper.

## Footnotes

1 The code for this study is publicly available at https://github.com/BMIRDS/3dMRISegmentation.
