# Supplementary material for: Automatic post-stroke lesion segmentation on MR images using 3D residual convolutional neural network
Source: Neuroimage Clin. 2020 May 26;27:102276. doi: 10.1016/j.nicl.2020.102276 (PMC7281812; doi:10.1016/j.nicl.2020.102276)
Supplement: Supplementary data 1 [file mmc1.docx]

**Supplementary Material**

**Appendix E1: Details of our 3D U-Net**

**Appendix E2: Objective functions**

**Appendix E3: Volume size selection in Zoom-In&Out training strategy**

**Figure E1: Additional segmentation results on the test set**

**Figure E2: Error analysis**

**Table E1: The distribution of primary stroke location in the dataset**

**Table E2: The average number of stroke** **lesions found in each area of the brain**

**Table E3. Data distribution by imaging site**

**Table E4. Data distribution by MRI scanner**

**Table E5. The list of subject IDs from the ATLAS dataset for each data split**

**Table E6. Summary of evaluation metrics based on the primary stroke locations**

**Table E7. Summary of evaluation metrics based on the vascular territories**

**Video E1: Visualization of segmentation results in a sequence of axial slices**

**Appendix E1: Details of our 3D U-Net**

We devised multiple methodological advancements and applied numerous modifications on the original 3D U-Net architecture based on the nature of our study. The first modification was designed to replace each convolutional layer in the network with a residual block to benefit from residual learning (1). In the case of down-sampling where the spatial size of the output tensor is different from the input in a residual block, we apply average pooling and zero-padding because they are non-parametric and thus do not cost in computer memory. Second, we use group normalization (GN) layers (2) instead of batch normalization (BN) layers (3) to stabilize the optimization with a small mini-batch size. Our volumetric segmentation task requires processing a sample of large volume, and, therefore, it is not feasible to process a large number of volumes at the same time, while BN is known for a faster convergence with a large (>= 32) mini-batch size. GN applies a normalization over a subset of channels rather than a mini-batch, so it works well even when the mini-batch size is small. This modification has shown successful results in another study on MRI volume (4). Furthermore, we apply a differentiable trilinear interpolation after each deconvolution operation to equalize the width and height of features between the endpoints of long skip connections. This technique enables the networks to work with any size of input volume without resizing as a pre-processing step. Our final model has 52 convolutional layers, as depicted in Figure 1.

To develop a network for the segmentation of other datasets, we suggest starting with our network configuration as a basis. Inserting more residual blocks, depicted as green boxes in Figure 1, after the ninth residual block at the center of the network can increase the capacity of the network and could potentially improve performance at the risk of overfitting. Inserting more blocks before down-sampling layers may help to detect small lesions, but would also requires more GPU memory. The same principle is applied to the number of convolutional filters in each block: increasing the filters, depicted as numbers on top of each block in Figure 1, may improve segmentation performance, and would also weigh more on GPU memory. Besides the details and hyperparameters of the optimization process listed in Table 1, we suggest increasing the length of training when fewer training samples are available or when the input volume size is small. Finally, scaling the initial learning rate at the zoom-in stage may be effective when mini-batch size increases (e.g., scaling the learning rate proportionally to the square root of the mini-batch size). On the other hand, reducing the initial learning rate at the zoom-out stage could be an option when overfitting is observed.

**References**

1. He K, Zhang X, Ren S, Sun J. Identity mappings in deep residual networks. European conference on computer vision 2016:630-645.

2. Wu Y, He K. Group normalization. Proceedings of the European Conference on Computer Vision (ECCV)2018; p. 3-19.

3. Ioffe S, Szegedy CJapa. Batch normalization: Accelerating deep network training by reducing internal covariate shift. 2015.

4. Myronenko A. 3D MRI brain tumor segmentation using autoencoder regularization. International MICCAI Brainlesion Workshop: Springer, 2018; p. 311-320.

**Appendix E2: Objective Functions**

The objective function, *L,* uses an affine combination of binary cross entropy loss (BCE) and soft Dice Coefficient loss (Dice):

$$L\left( Y, \hat{Y} \right)=\alpha BCE\left( Y, \hat{Y} \right)+(1-\alpha)Dice(Y, \hat{Y})$$

$Y\in{\{0,1\}}^{N}$ is a segmentation label and $\hat{y}={[0,1]}^{N}$ is the output for an *N*-voxel input. BCE and Dice are defined as:

$$BCE\left( Y, \hat{Y} \right)= -\frac{1}{N}\sum_{i=0}^{N} \beta Y_{i}\log\hat{Y}_{i}+(1-\beta)(1-Y_{i})\log1-\hat{Y}_{i}$$

and

$$Dice\left( Y, \hat{Y} \right)= \frac{2\sum_{i=0}^{N} Y_{i}\hat{Y}_{i}}{\sum_{i=0}^{N} {Y_{i}}^{2}+\sum_{i=0}^{N} {\hat{Y}_{i}}^{2}}$$

In this objective function, we used $\alpha=0.1$ and $\beta=0.9967$.

**Appendix E3: Volume size selection in Zoom-In&Out training strategy**

We use a two-stage training strategy to utilize our asymmetric configuration of graphic processing units (GPUs). In our experiments, the input volume size at each stage was determined by two factors: the capacity of GPU memory and the observed range of lesion size in each dimension. In the zoom-in stage, we used the largest possible cubic volume that fits in a GPU with smaller memory capacity in order to learn volumetric features. In the zoom-out stage, we used a GPU with a larger memory capacity to calibrate our segmentation model with a broader spatial context. Each dimension of input volume size was adjusted to enclose all the observed lesions in the training set, increasing the likelihood to achieve accurate segmentation of scans in one shot.

Deciding input volume size in each stage for a dataset depends mainly on the original image volume and target lesion size, in addition to the hardware environment. To apply our training strategy to other datasets, first, we suggest visualizing typical images from the dataset, and then reviewing the spatial distribution of target lesions to estimate the possible effective input volume size for a specific application. In our study, we observed in early experiments that using volume sizes significantly smaller than those in our final configuration does not achieve a competitive performance. This performance gap may indicate that volumes cropped for training during these experiments were overwhelmed by regions without positive stroke lesions, which could have negatively biased our model optimization in the early experiments. We also suggest performing a systematic search on proportions of original volume size (e.g., 25%, 50%, 75% ) to find an optimal configuration.

**Figure E1. Additional segmentation results on the test set**


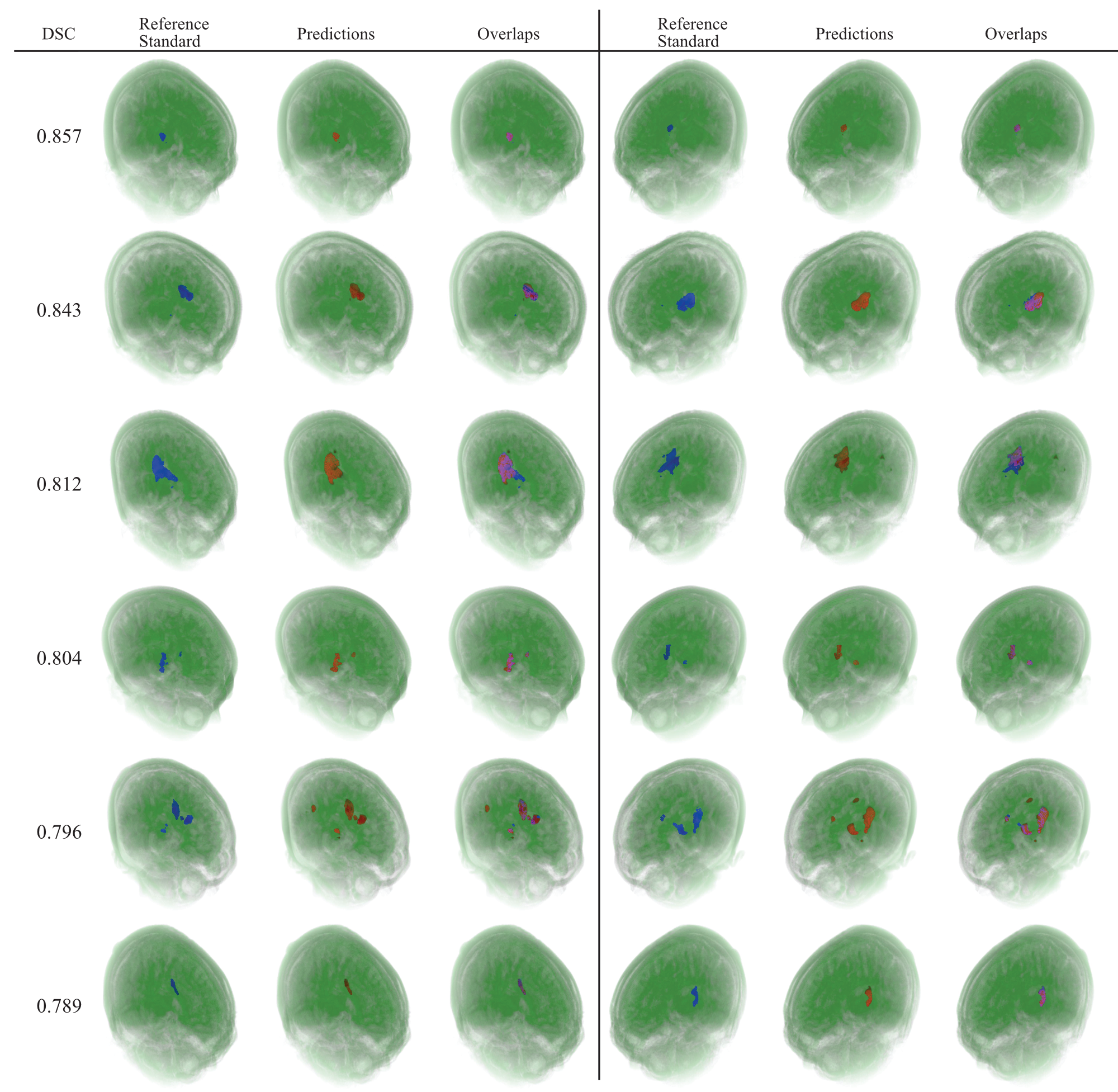


Figure E1. Additional visualizations of test samples in the same fashion as in Figure 2. The third and sixth columns present the overlap between the reference standard labels and predictions.

**Figure E2. Error Analysis**


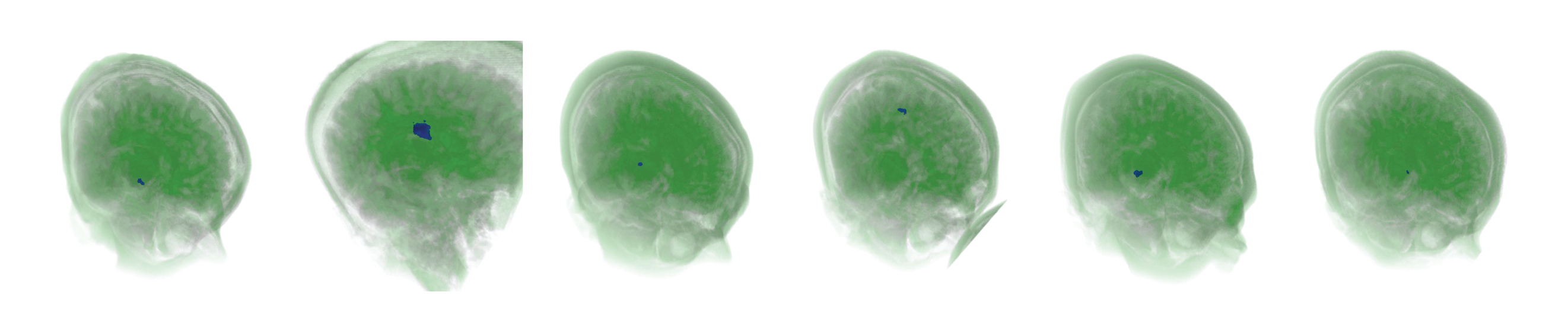


Figure E2. Samples with the worst DSCs. Among them, five samples have very small lesions and one sample (the second from left) seems to have spatially different characteristics from others. This discrepancy might be due to an error in scanning or the normalization process. We also observed a few of such discrepancies in our training and development datasets.

**Table E1.** **The distribution of primary stroke location in the dataset**

|  | Primary Stroke Location | | | |
| --- | --- | --- | --- | --- |
| Dataset | Left | Right | Brainstem | Bilateral |
| training | 91 | 80 | 10 | 1 |
| development | 14 | 9 | 2 | 1 |
| test | 22 | 6 | 2 | 1 |
| All | 127 | 95 | 14 | 3 |

**Table E2.** **The average number of stroke** **lesions found in each area of the brain**

|  | The Number of Strokes per Case | | | | |
| --- | --- | --- | --- | --- | --- |
| Dataset | Left Cortical | Left Subcortical | Right Cortical | Right Subcortical | Other |
| training | 0.16±0.42 | 0.69±0.83 | 0.17±0.39 | 0.57±0.71 | 0.14±0.36 |
| development | 0.35±0.64 | 0.46±0.65 | 0.19±0.50 | 0.69±1.03 | 0.23±0.44 |
| test | 0.13±0.34 | 0.84±0.73 | 0±0 | 0.77±0.84 | 0.10±0.30 |
| All | 0.18±0.44 | 0.69±0.80 | 0.15±0.38 | 0.61±0.76 | 0.14±0.36 |

**Table E3.** **Data distribution by imaging site**

|  | training | validation | test |
| --- | --- | --- | --- |
| c0003 | 42 | 3 | 10 |
| c0004 | 20 | 7 | 7 |
| c0005 | 21 | 2 | 5 |
| c0006 | 11 | 0 | 1 |
| c0007 | 22 | 4 | 4 |
| c0008 | 14 | 1 | 0 |
| c0009 | 8 | 0 | 3 |
| c0010 | 31 | 8 | 1 |
| c0011 | 13 | 1 | 0 |
| Total | 182 | 26 | 31 |

**Table E4.** **Data distribution by MRI scanner**

|  | training | validation | test |
| --- | --- | --- | --- |
| GE 750 Discovery | 56 | 9 | 4 |
| GE Signa Excite | 20 | 7 | 7 |
| GE Signa HD-X | 17 | 2 | 7 |
| Phillips Achieva | 22 | 4 | 4 |
| Siemens Trio | 67 | 4 | 9 |
| Total | 182 | 26 | 31 |

**Table E5. The list of subject IDs from the ATLAS dataset for each data split**

| Training set | c0003s0001t01 c0003s0050t01 c0005s0030t01 c0007s0032t01 c0010s0013t02  c0003s0003t01 c0003s0051t01 c0005s0031t01 c0007s0033t01 c0010s0014t01  c0003s0004t01 c0003s0053t01 c0005s0035t01 c0007s0034t01 c0010s0015t01  c0003s0005t01 c0003s0055t01 c0005s0036t01 c0007s0035t01 c0010s0015t02  c0003s0006t01 c0003s0056t01 c0005s0040t01 c0007s0036t01 c0010s0020t01  c0003s0007t01 c0004s0001t01 c0005s0042t01 c0008s0001t01 c0010s0020t02  c0003s0008t01 c0004s0002t01 c0005s0045t01 c0008s0002t01 c0010s0021t01  c0003s0009t01 c0004s0003t01 c0005s0047t01 c0008s0003t01 c0010s0024t01  c0003s0010t01 c0004s0004t01 c0005s0049t01 c0008s0006t01 c0010s0025t01  c0003s0012t01 c0004s0005t01 c0006s0001t01 c0008s0008t01 c0010s0025t02  c0003s0013t01 c0004s0008t01 c0006s0003t01 c0008s0009t01 c0010s0028t01  c0003s0014t01 c0004s0010t01 c0006s0004t01 c0008s0010t01 c0010s0031t01  c0003s0015t01 c0004s0013t01 c0006s0005t01 c0008s0011t01 c0010s0032t01  c0003s0016t01 c0004s0014t01 c0006s0006t01 c0008s0014t01 c0010s0034t01  c0003s0017t01 c0004s0016t01 c0006s0008t01 c0008s0028t01 c0010s0037t01  c0003s0018t01 c0004s0018t01 c0006s0010t01 c0008s0029t01 c0010s0040t01  c0003s0019t01 c0004s0019t01 c0006s0014t01 c0008s0031t01 c0010s0041t01  c0003s0020t01 c0004s0021t01 c0006s0018t01 c0008s0032t01 c0010s0042t01  c0003s0021t01 c0004s0024t01 c0006s0019t01 c0008s0033t01 c0010s0043t01  c0003s0023t01 c0004s0025t01 c0006s0020t01 c0009s0002t01 c0010s0044t01  c0003s0024t01 c0004s0027t01 c0007s0001t01 c0009s0005t01 c0010s0044t02  c0003s0026t01 c0004s0028t01 c0007s0002t01 c0009s0006t01 c0011s0001t01  c0003s0027t01 c0004s0029t01 c0007s0003t01 c0009s0007t01 c0011s0002t01  c0003s0028t01 c0004s0032t01 c0007s0005t01 c0009s0009t01 c0011s0003t01  c0003s0029t01 c0004s0034t01 c0007s0010t01 c0009s0010t01 c0011s0004t01  c0003s0030t01 c0005s0003t01 c0007s0012t01 c0009s0011t01 c0011s0006t01  c0003s0031t01 c0005s0007t01 c0007s0013t01 c0009s0012t01 c0011s0007t01  c0003s0034t01 c0005s0008t01 c0007s0015t01 c0010s0002t02 c0011s0008t01  c0003s0036t01 c0005s0010t01 c0007s0017t01 c0010s0003t02 c0011s0010t01  c0003s0037t01 c0005s0012t01 c0007s0019t01 c0010s0005t01 c0011s0011t01  c0003s0038t01 c0005s0013t01 c0007s0020t01 c0010s0005t02 c0011s0012t01  c0003s0039t01 c0005s0014t01 c0007s0025t01 c0010s0006t01 c0011s0013t01  c0003s0043t01 c0005s0018t01 c0007s0026t01 c0010s0009t01 c0011s0014t01  c0003s0044t01 c0005s0021t01 c0007s0027t01 c0010s0009t02 c0011s0015t01  c0003s0045t01 c0005s0024t01 c0007s0028t01 c0010s0010t01  c0003s0046t01 c0005s0028t01 c0007s0029t01 c0010s0011t02  c0003s0049t01 c0005s0029t01 c0007s0031t01 c0010s0013t01 |
| --- | --- |
| Development set | c0003s0002t01 c0004s0012t01 c0007s0014t01 c0010s0016t02 c0010s0038t01  c0003s0042t01 c0004s0020t01 c0007s0018t01 c0010s0018t01 c0011s0009t01  c0003s0054t01 c0004s0031t01 c0007s0022t01 c0010s0022t01  c0004s0006t01 c0004s0033t01 c0007s0024t01 c0010s0022t02  c0004s0009t01 c0005s0017t01 c0008s0005t01 c0010s0029t01  c0004s0011t01 c0005s0027t01 c0010s0016t01 c0010s0030t01 |
| Test set | c0003s0011t01 c0003s0041t01 c0004s0023t01 c0005s0048t01 c0009s0004t01  c0003s0022t01 c0003s0048t01 c0004s0026t01 c0006s0007t01 c0009s0008t01  c0003s0025t01 c0003s0052t01 c0004s0030t01 c0007s0016t01 c0010s0046t01  c0003s0032t01 c0004s0007t01 c0005s0006t01 c0007s0021t01  c0003s0033t01 c0004s0015t01 c0005s0009t01 c0007s0023t01  c0003s0035t01 c0004s0017t01 c0005s0011t01 c0007s0030t01  c0003s0040t01 c0004s0022t01 c0005s0026t01 c0009s0003t01 |

**Table E6.** **Summary of evaluation metrics based on the primary stroke locations**

| **Primary Stroke Locations (N)** | **DSC** | **HD (mm)** | **ASSD (mm)** | **TPR** | **Precision** |
| --- | --- | --- | --- | --- | --- |
| Left (22) | 0.65 (0.49-0.78) | 23.5 (10.4-38.4) | 4.1 (1.7-7.3) | 0.78 (0.62-0.90) | 0.63 (0.45-0.77) |
| Right (6) | 0.70 (0.40-0.85) | 9.8 (1.7-27.0) | 2.1 (0.3-6.7) | 0.85 (0.77-0.95) | 0.68 (0.33-0.89) |
| Brainstem (2) | 0.39 (0.32-0.46) | 27.2 (5.4-49.0) | 4.9 (1.5-8.3) | 0.91 (0.83-0.99) | 0.25 (0.19-0.32) |
| Bilateral (1) | 0.79 (0.79-0.79) | 4.6 (4.6-4.6) | 1.3 (1.3-1.3) | 0.88 (0.88-0.88) | 0.72 (0.72-0.72) |

N: the number of subjects in each set.

**Table E7.** **Summary of evaluation metrics based on the Vascular Territories**

| **Vascular Territories (N)** | **DSC** | **HD (mm)** | **ASSD (mm)** | **TPR** | **Precision** |
| --- | --- | --- | --- | --- | --- |
| Lacunar (22) | 0.69  (0.58-0.79) | 19.6  (7.4-34.2) | 3.2  (1.3-5.8) | 0.81 (0.67-0.91) | 0.69 (0.55-0.80) |
| MCA (5) | 0.65  (0.26-0.85) | 22.2  (4.9-54.8) | 5.4  (1.5-13.6) | 0.89 (0.82-0.96) | 0.69 (0.24-0.81) |
| Basilar perforator (3) | 0.26  (0.00-0.46) | 27.2  (5.4-49.0) | 4.9  (1.5-8.3) | 0.61 (0.00-0.99) | 0.17 (0.00-0.32) |
| Anterior choroidal (1) | 0.68  (0.68-0.68) | 15.3  (15.3-15.3) | 2.2  (2.2-2.2) | 0.87 (0.87-0.87) | 0.56 (0.56-0.56) |

N: the number of subjects in each set.

**Video E1: Visualization of segmentation results in a sequence of axial slices**

Files: Video_E1_a.mov, Video_E1_b. mov, Video_E1_c. mov, Video_E1_d. mov, Video_E1_e. mov, Video_E1_f. mov

Each video is the visualization of segmentation results on selected scans in Figure 2, projecting lesion predictions (in red) and reference labels (in blue) onto a sequence of axial slices (in green). Purple color represents the regions where prediction and the reference agree. Video_E1_a.mov to Video_E1_c.mov correspond to samples on the left and Video_E1_d.mov to Video_E1_f.mov correspond to those on the right in Figure 2.
